# Rotational photonic spin Hall effect

**DOI:** 10.1515/nanoph-2023-0559

**Published:** 2023-11-08

**Authors:** Yougang Ke, Yongfeng Bian, Qiang Tang, Jibo Tian, Linzhou Zeng, Yu Chen, Xinxing Zhou

**Affiliations:** School of Information Science and Engineering, Hunan Institute of Science and Technology, Yueyang 414006, China; International Collaborative Laboratory of 2D Materials for Optoelectronics Science and Technology, Institute of Microscale Optoelectronics, Shenzhen University, Shenzhen 518060, China; Key Laboratory of Low-Dimensional Quantum Structures and Quantum Control of Ministry of Education, Synergetic Innovation Center for Quantum Effects and Applications, School of Physics and Electronics, Hunan Normal University, Changsha 410081, China

**Keywords:** photonic spin Hall effect, spin-orbit interaction, Pancharatnam–Berry phase, metasurface, spin-Hall device

## Abstract

Multidimensional manipulation of photonic spin Hall effect (PSHE) has attracted considerable interest due to its potential in a wide variety of spin-based applications. Plenty of research efforts have been devoted to transverse or longitudinal spin-dependent splitting; however, the splitting pattern that can self-rotate in a three-dimensional (3-D) space appears to be missing in literature. In this paper, we introduce a novel 3-D rotational PSHE, which can be realized and tuned using well-designed Pancharatnam–Berry phase metasurfaces. To demonstrate this phenomenon, we first show that when a single dielectric metasurface is used, the lobe-structured spin-splitting patterns on the transverse planes rotate and evolve along the propagation path. Then, we present that under two cascaded metasurfaces, the rotation angle of the splitting patterns are tunable by adjusting the relative rotation angle between the two metasurfaces. Finally, we manifest that the lobe number of the two spin-dependent splitting patterns can be independently controlled once we introduce a dynamic phase, which produces an asymmetrical rotational PSHE. The demonstrated phenomena can be used to achieve active manipulation of spin photons in multiple dimensions, and the developed device might find potential applications in various areas, e.g., optical microscopy.

## 1 Introduction

Photonic spin Hall effect (PSHE) originates from the spin–orbit interaction of light, which exhibit itself as the spin-dependent splitting in the space [1–3]. Normally, the spin-dependent splitting created by the PSHE in light deflection or refraction at an optical interface between two different media is limited to a fraction of one wavelength, due to very weak spin–orbit interaction. The polarization degree of freedom approach can be used to magnify the maximum separation in the PSHE into dozens of wavelengths, and the dielectric grating method is able to strengthen the PSHE to several wavelengths [4, 5]. The direct observation of the PSHE is challenging, which necessitates the use of quantum weak measurement technology for this tiny spin-dependent splitting to be observed [6]. Since the PSHE is very sensitive to the optical parameters of interface materials, PSHE combining the quantum weak measurement provides a precision metrology tool to characterize optical parameters [7–10]. The tiny spin-dependent splitting is also exploited in optical differential operation and image edge detection [11, 12].

To manipulate the spin–orbit interaction in a desired manner, optical metasurfaces, which are essentially two-dimensional optical metamaterials with planar and ultrathin nanostructures, have been considered as a new paradigm to achieve a stronger or larger spin-dependent splitting in PSHE [13–15]. Among the different types of optical metasurfaces, all-dielectric metasurfaces provide a high efficiency in transmission mode compared to plasmonic counterparts [16]. Both the flexible splitting manipulation and the high transmission efficiency are crucial to PSHE-based applications [17, 18]. In the past few years, many metasurface-based PSHE devices have been proposed such as metalenses [19–21], beam deflectors [22–24], spin-dependent holograms [25, 26], and photonic spin filters [27, 28]. More recently, the PSHE finds potential application scenarios in on-chip high-speed coherent optical signal detection and visual cryptography [29, 30]. Since realizing arbitrarily manipulation of the spin separation in PSHE is significant to PSHE-based applications, enormous works focusing on the manipulation of PSHE based on the metasurfaces have been reported. However, most of them have been merely devoted to transverse or longitudinal spin-dependent splitting. In the traditional PSHE, the two spin states split from each other in a fixed direction, and only the splitting displacement is a controllable variable. Very recently, the oscillated PSHE has been proposed and realized [31, 32], which provides a method to control the trajectories of the separated spin states in a periodically regulated way. Different from the traditional PSHE, in the oscillated PSHE, the spin states intertwine (for the spiral-like subclass), or cross (for the cosine-like subclass) each other periodically. However, the incident beam is simply separated into two light spots that remain the respective shape during the propagation as it originally is, and moreover, the transverse profiles vary periodically, which causes uncertainty for us to judge the propagation distance from the transverse profile. More importantly, the intertwining or crossing nature of the two spin states increases the difficulty in identifying the current status of the oscillatory PSHE in propagation, especially if disturbance exists.

To address the aforementioned issues, in this paper, we propose a tunable rotational 3-D PSHE that can be achieved by well-designed all-dielectric metasurfaces. Compared to the oscillated PSHE, the proposed rotational PSHE is shown to have the following advantages: (1) The spin-dependent splitting not only exists in the transverse direction but also manifests itself as two self-rotating patterns along the propagation direction. By utilizing the displacement magnitude together with the rotation angle, one can reversely derive the propagation distance from the transverse intensity in a more accurate way. (2) In the oscillatory PSHE, the incident beam is simply separated into two plain spots, whose uniformity is prone to disturbance during long-distance propagation. In contrast, the two blade-like patterns in the proposed rotational PSHE have more intricate internal structures that look like lobes, which are endowed with a stronger ability to resist the disturbance. (3) When using two cascaded optical elements, one can alter the number of lobes by changing the polarization state of the incident light and control the angle of rotation by adjusting the relative rotation angle of the two elements. Moreover, by introducing the dynamic phase to the phase design, independent manipulation of the two opposite spin states can be realized, which enables us to implement different numbers of lobes on the two patterns. The relative rotational angle between the two different spin-dependent patterns is also dynamically controllable, which further improved the controllability of the spin photons.

**Figure 1:**
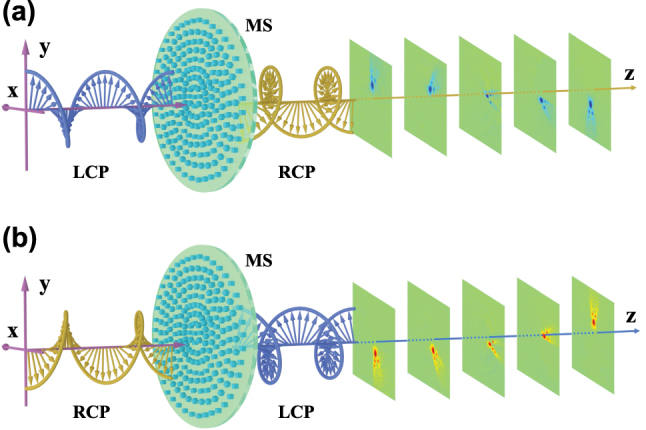
Schematic illustration of the generation of rotational spin-dependent splitting based on the Pancharatnam–Berry phase metasurface (MS). Rotational directions of the generated beams are controlled by the handedness of the incident beams: (a) a LCP incident case and (b) a RCP incident case.

As depicted in Figure 1, if a circularly polarized beam impinges normally to the metasurfaces, the transmitted beam self-rotates along the propagation path. The rotational direction is governed by the handedness of the incident beam. If the incident beam is linearly polarized, the spin-dependent splitting is created in the transmitted beam, and the corresponding splitting patterns rotate and evolve in transverse plane along the propagation path. The underlying physics is associated with the Pancharatnam–Berry geometrical phases, which can be visualized on the Poincaré sphere [33, 34]. The additional Pancharatnam–Berry geometrical phases of the transmitted circularly polarized light are ±2*α*. Here *α* is the orientation angle of the local optical axis in the metasurface, which determines the polarization evolving routes on the surface of the Poincaré sphere. The sign of an additional Pancharatnam–Berry geometrical phase depends on the polarization evolving directions on the Poincaré sphere, + for evolving from the north to the south poles and – for evolving from the south to the north poles. Thereby, by designing the patterns of the space-varying optical axis, a desired metasurface-based Pancharatnam–Berry phase element shall be obtained [35–37], which can be used to realize the following phenomenon: *the two spin components splitting in angular direction while along the propagation path experiencing self-rotation in opposite directions*. This is our definition for the rotational PSHE.

In the traditional PSHE, the beam usually separates into two simple symmetrical circular light spots. However, from the perspective of self-rotating beams, the directionality of the circular light spots under different spin states is comparatively weak, and the uniformity of such beams is prone to disturbance during long-distance propagation. To resolve this issue, some researchers have proposed new kinds of PSHE that produce non-symmetrical light spots. In Ref. [38], by breaking the rotational symmetry of local optical axes, Liu et al. observed non-symmetrical spin-dependent splitting in the far field. In addition, Zhang et al. demonstrated the controllability of such splitting [39]. From these works, it can be seen that the PSHE is clearer when each of the spin states manifests itself as non-symmetrical light patterns. As a result, we choose the non-symmetrical blade-like pattern. Blade-like beam breaking the rotation symmetry of intensity of optical field allows us to achieve a complete separation of the intensity of the LCP and RCP components. Besides, blade-like beam exhibits non-diffraction and self-healing properties, which is suitable for information transmission. During long-distance propagation, the blade-like pattern is less vulnerable to environmental disturbance, which reduces the error rate of information transmission.

Although the blade-like patterns used in this study have already been generated and analyzed by the metasurface communities, e.g., in Refs. [40, 41], these papers focused on the generation and properties of the beam as a whole, which paid few attention to the separation of the two spin components. Compared to [40], the novelty of this study is the rotatability of the two blade-like patterns along the propagation path. Meanwhile, compared to [41], the impact of this work lies in the beam splitting into two spin-dependent patterns. Different from those previously reported works on PB phase-based phenomena, this work concentrates on the controllable behavior of the splitting patterns rather than the generation of the beam alone.

The remainder of the paper is organized as follows. Section 2 describes the theoretical models. Section 3 presents theoretical and simulation results, as well as some discussions on those results. Finally, conclusions are drawn in Section 4.

## 2 Theoretical models

### 2.1 Propagation of the beam passing through the Pancharatnam–Berry phase metasurface

Realizing the rotational PSHE requires the design of metasurfaces for this specific purpose. Suppose a Gaussian beam with an arbitrary homogeneous polarization state normally impinge onto a Pancharatnam–Berry phase metasurface. The polarization state geometrically corresponds to a point on the surface of the Poincaré sphere, which can be algebraically represented in terms of its polar angle *ϑ* and azimuthal angle *ϕ* [42]. The electric fields of the incident beam can be expressed as [43]

(1) $$E_{\;\text{in}}\left(x,y,0\right)=E_{\text{G}}\left(x,y\right)\left(\text{cos}\vartheta {\text{e}}^{-\text{i}\phi }\left|L\right\rangle +\sin\vartheta {\text{e}}^{\text{i}\phi }\left|R\right\rangle \right).$$

Here, $E_{\text{G}}\left(x,y\right)=E_{0}\text{exp}\left(-\frac{x^{2}+y^{2}}{\omega_{0}^{2}}\right)$ is the amplitude of the incident beam and *ω*_0_ is the beam waist. $\left|L\right\rangle =\left({\vec{e}}_{x}+i{\vec{e}}_{y}\right)/\sqrt{2}$ and $\left|R\right\rangle =\left({\vec{e}}_{x}-i{\vec{e}}_{y}\right)/\sqrt{2}$ represent the LCP and RCP, respectively. The Jones matrix of the Pancharatnam–Berry phase metasurface (the birefringent phase retardation of *π* and local direction of optical axis specified by *α*) can be written as [44]

(2) $$T\left(x,y\right)=\left(\begin{array}{cc} \cos2\alpha & \sin2\alpha \\ \sin2\alpha & -\cos2\alpha \end{array}\right).$$

Besides, the electric fields of beam emerging from the element can be obtained as

(3) $$\begin{array}{cc} E_{\text{out}}\left(x,y,0\right) & =T\left(x,y\right)E_{\;\text{in}}\left(x,y,0\right)=E_{\text{G}}\left(x,y\right)\cdot \\ & \;\times \left[\text{sin}\vartheta {\text{e}}^{-\text{i}\left(2\alpha -\phi \right)}\left|L\right\rangle +\cos\vartheta {\text{e}}^{\text{i}\left(2\alpha -\phi \right)}\left|R\right\rangle \right]. \end{array}$$

Equations (1) and (3) show that the Pancharatnam–Berry phase element inverts the handedness of normally incident photons. Meanwhile, the handedness-reversed photons obtain a spin- and position-dependent additional phase $\pm 2\alpha \left(x,y\right)$ depending on the patterns of space-variant optical axis orientations, where + is for LCP beam incidence and – is for RCP beam incidence.

The diffraction field in the Fresnel region can be calculated via the Fresnel diffraction integral formula [45], which is given by

(4) $$\begin{array}{cc} E_{\text{diff}}\left(x,y,z\right) & =\frac{\text{exp}\left(i2\pi z/\lambda \right)}{i\lambda z}\overset{+\infty }{\underset{-\infty }{\iint }}E_{\text{out}}\left(x^{\prime },y^{\prime },0\right)\cdot \\ & \;\times \text{exp}\left(i\frac{\pi }{\lambda z}\left[{\left(x-x^{\prime }\right)}^{2}+{\left(y-y^{\prime }\right)}^{2}\right]\right)dx^{\prime }dy^{\prime }\;\;. \end{array}$$

To characterize the separation of spin photons in PSHE, the normalized Stokes parameter *S*_3_ is employed, which can be expressed as [46]

(5) $$S_{3}=\frac{2\left|E_{\text{diff}}^{x}\left(x,y,z\right)\right|\left|E_{\text{diff}}^{y}\left(x,y,z\right)\right|\text{sin}\left(\Phi_{x}-\Phi_{y}\right)}{{\left|E_{\text{diff}}^{x}\left(x,y,z\right)\right|}^{2}+{\left|E_{\text{diff}}^{y}\left(x,y,z\right)\right|}^{2}},$$

where *Φ*_*x*_ and *Φ*_*y*_ are the phases of the diffraction fields $E_{\text{diff}}^{x}\left(x,y,z\right)$ and $E_{\text{diff}}^{y}\left(x,y,z\right)$ , respectively.

### 2.2 Design of the Pancharatnam–Berry phase metasurface

**Figure 2:**
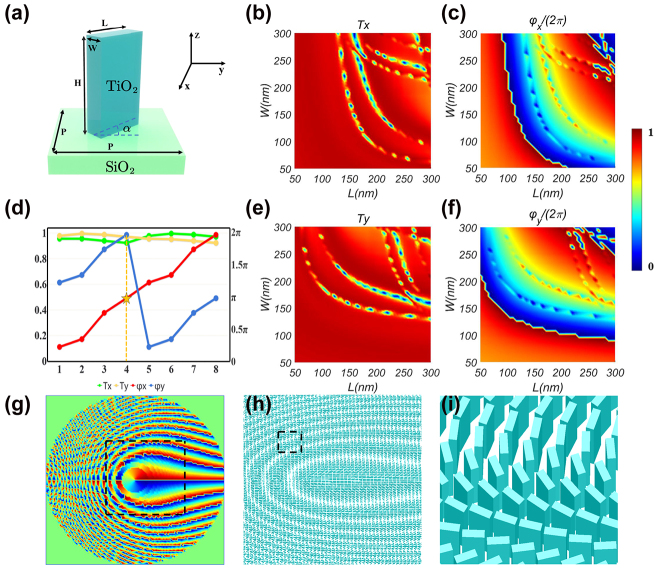
Schematic summary of the metasurface design process. (a) Perspective view and the structural parameters of the unit cell of the dielectric metasurface. (b) and (c) The simulated transmission coefficient and phase shift as a function of varied length and width for *x*-polarized incident light. (e) and (f) The simulated transmission coefficient and phase shift for the *y*-polarized incident case. (d) The corresponding transmission coefficients and phase shifts of the selected 8 nanofins. (g) The designed phase for rotational PSHE. (h) The designed metasurface corresponding to the phase selected by the black dotted box in (g). (i) The enlarged selected area in the black dotted square in (h).

Schematics of the metasurface design process are depicted in Figure 2. The adopted unit cell of the dielectric metasurface consists of a rectangular TiO_2_ nanofin with a fixed height of *H* = 600 nm embedded on a square SiO_2_ substrate with the lattice constant *P* = 340 nm (see Figure 2(a)). Here TiO_2_ is chosen due to its high refractive index and low loss in the visible range [47]. To determine the parameters (the length and width of the rectangular TiO_2_ nanofin), the finite-difference time-domain (FDTD) method is performed with the wavelength of incident plane wave as *λ* = 532 nm. In the simulations, periodic boundary conditions are used in the *x* and *y* directions, and perfectly matched layers are applied in the *z* direction.

Under the *x*-polarized illumination, the simulated transmission coefficient (*T*_*x*_) and phase shift (*φ*_*x*_) as a function of varied length and width are shown in Figure 2(b) and (c). Under the *y*-polarized illumination, the simulated transmission coefficient (*T*_*y*_) and phase shift (*φ*_*y*_) are presented in Figure 2(e) and (f). From Figure 2(b)–(e), we can find that the simulated unit cells have high efficiency except few combination of the length *L* and width *W* of the rectangular TiO_2_ nanofin. These points with low efficiency should be skipped in building highly efficient metasurface devices. Figure 2(c)–(f) show that *φ*_*x*_ and *φ*_*y*_ cover the entire interval of [0, 2*π*] and can be independently controlled by the parameters (the length and width of nanofin). Thereby, an arbitrary phase difference Δ*φ* = *φ*_*x*_ − *φ*_*y*_ ranging from 0 to 2*π* can be achieved by properly choosing the length (*L*) and the width (*W*) of the nanofin. The transmittances (*T*_*x*_ and *T*_*y*_) and phase shifts (*φ*_*x*_ and *φ*_*y*_) of eight selected nanofins are plotted in Figure 2(d). The transmittances of the eight different nanofins are close to 1, and the phase difference Δ*φ* = *φ*_*x*_ − *φ*_*y*_ approaches *π*. Hence, each selected nanofin can be regarded as a half-wave plate. On the whole, the metasurface is a planar photonic device composed of such nanostructures, which works as an array of spatially variable half-wave plates. Each of the nanofins has its own orientation according to its spatial location. Compared to metallic metasurfaces, the suggested all-dielectric metasurface has high transmission efficiency without suffering from the Ohmic losses.

Here, the fourth nanofin is selected as the basic element of the metasurface, and the corresponding length and width are 300 nm and 110 nm, respectively. Generally speaking, the working principle of the metasurface is based on the shift of the wave vector, which results from the geometric Pancharatnam–Berry (PB) phase. On the metasurface, there are regularly arranged nanofins, each of which has a certain spatial orientation angle. When the light beam passes through a nanofin, the geometric Pancharatnam–Berry (PB) phase will be induced. The geometric PB phase is related to the spatial orientation angle of the nanofin, and has opposite signs for opposite spin states (i.e., LCP and RCP). Due to the PB phase, a shift of the wave vector occurs in the momentum space, which results in a real space shift of light. This will be observed as a spin-dependent splitting, namely the PSHE. By rotating the nanofin, arbitrary Pancharatnam–Berry phase patterns can be achieved. Meanwhile, by resizing the nanofin, the dynamic phase can also be introduced. If all the eight selected nanofins are used in the metasurface design, independent control of the two splitting patterns can be realized in such a Pancharatnam–Berry phase and dynamic phase combined way. This new phenomenon will be referred to as the *asymmetric* rotational PSHE.

## 3 Results and discussion

**Figure 3:**
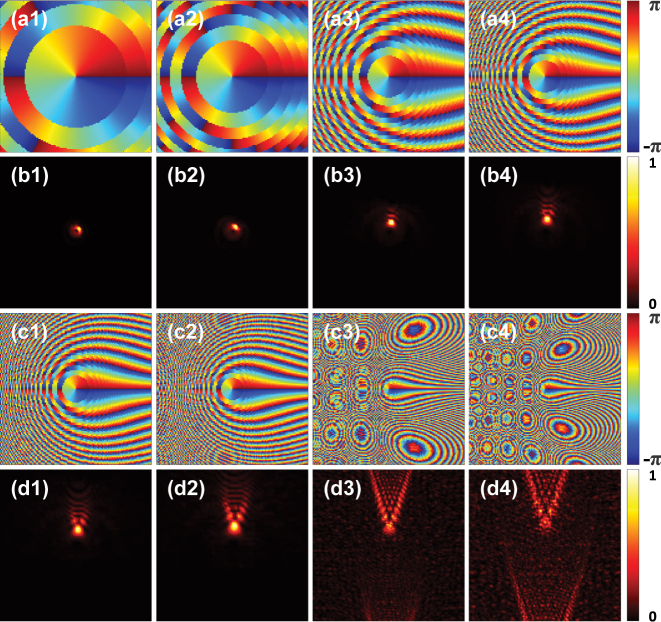
The phase profiles and beam intensities for different values of *a* under the LCP incidence, where the parameter *b* is fixed at *b* = 2. (a1)–(a4) and (c1)–(c4) The phase profiles for *a* = 5000 × 10^6^, 10,000 × 10^6^, 30,000 × 10^6^, 50,000 × 10^6^, 70,000 × 10^6^, 101,115 × 10^6^, 401,115 × 10^6^, 601,115 × 10^6^. (b1)–(b4) and (d1)–(d4) The corresponding beam intensities.

In this work, to realize the rotational PSHE, the continuous phase function *ψ* is used [48]:

(6) $$\psi \left(r,\theta \right)=\pm \text{ceil}\left(a\cdot r^{b}\right)\cdot \theta ,$$

where the parameter *a* controls the beam tail and *b* adjusts the phase profile. In this paper, we set *b* = 2, because the lobe-like patterns are comparatively clear and complete under this value [49]. To determine the parameter *a*, we show in Figure 3 the beam intensities and the phase distributions for different values of *a*. It can be seen that, with the increase of the value of *a*, the intensity spot gradually morphs into a blade-like pattern. However, when *a* reaches 401,115 × 10^6^ or over, the intensity of the main lobe will decrease, and noisy dots will appear around the pattern. This is caused by insufficient sampling as we only use a 122 × 122 metasurface. Under the circumstance, we choose *a* = 101,115 × 10^6^. Note that this value is much larger than the one used in Refs. [49, 50]. Again, this is because the sampling points in our metasurface are only 122 × 122. The symbol $\text{ceil}\left(\cdot \right)$ denotes the ceiling function that returns the largest integer that is smaller than or equal to the input value (i.e., rounds down the nearest integer). As for the sign ± wherein, + and – stands for LCP and RCP incidence, respectively. The additional phase distribution for a LCP is illustrated in Figure 2(g).

According to Eq. (3), the local direction of optical axis of the Pancharatnam–Berry phase metasurface is

(7) $$\alpha \left(r,\theta \right)=\text{ceil}\left(a\cdot r^{b}\right)\cdot \theta \;\;/2.$$

Presented in Figure 2(h) are distributions of the unit cell of the designed metasurface segment corresponding to phase selected by the black dotted box in Figure 2(g). Figure 2(i) shows the enlarged basic element distributions of the selected area in the black dotted square in Figure 2(h). We can see from Figure 2(i) that all basic elements of the metasurface have the same size but different rotation angles. It means that we can realize the local optical axis pattern governed by Eq. (7) by simply rotating the angle of unit cell.

**Figure 4:**
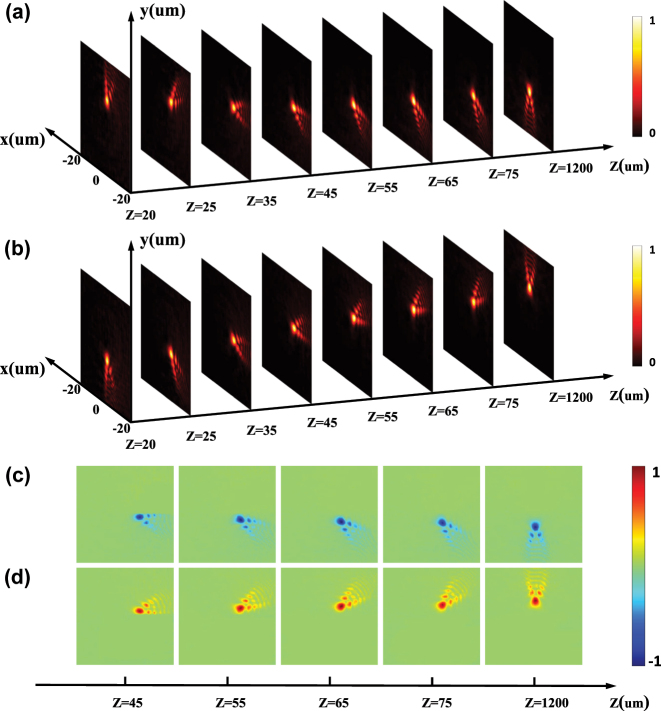
The calculated evolution intensity and Stokes parameters *S*_3_ of the beams behind the metasurface. (a) and (b) The evolving intensities under the LCP and RCP, respectively. (c) and (d) The Stokes parameters *S*_3_ corresponding to the beams on the selected transverse planes in (a) and (b), respectively. The metasurface inverts the handedness of the incident beams, and transmitted beams rotate respectively in clockwise and counter-clockwise directions along the propagation controlled by the handedness of the illumination beams.

To verify the principle of rotational PSHE, we first let a LCP Gaussian beam and a RCP one normally illuminate the metasurface, respectively. The normalized beam intensity and Stokes *S*_3_ of the beams on the selected transverse planes behind the metasurface are shown in Figure 4. Here the calculated results are based on the Fresnel diffraction integral formula as in Eq. (4). Figure 4(a) and (b) show that transmitted light beams become fan-shaped lobes. Figure 4 shows that the metasurface inverts the handedness of the incident beams, and the transmitted beams rotate, respectively, in the clockwise and counter-clockwise directions along the propagation for illumination by circularly polarized light beams of the opposite handedness.

As shown above, the designed metasurface can be used to realize the rotational PSHE. This interesting self-rotating beam has already been demonstrated in Refs. [49, 50]. The differences between this work and Refs. [49, 50] are summarized in the following aspects. (1) Principle: The underlying physics of the works reported in Refs. [49, 50] is associated with the dynamic phase. This work, by contrast, involves the geometrical phase as well as the optical integration of the Pancharatnam–Berry phase and the dynamical phase, which provide an additional degree of freedom for the manipulation of light. (2) Realization: The references [49, 50] were talking about a class of self-rotating beam that is generated by a single spatial light modulator, while this work is discussing the rotational spin-dependent splitting patterns produced by a dielectric metasurface. Although the spatial light modulator can be used to generate the self-rotating beams, it is difficult to use such a device to modulate independently LCP and RCP components of the incident beam, which is therefore not suitable for realizing the PSHE. In contrast, the single-layered dielectric metasurface used in our work allows us to encode two independent phase distributions to two orthogonal circular polarizations conveniently. (3) Application: Compared to the spatial light modulator used in Refs. [49, 50], the proposed metasurface has higher efficiency, lower cost, and smaller size, which can thus be easily integrated into other standard optical components for practical applications with miniaturization trends of optical devices.

**Figure 5:**
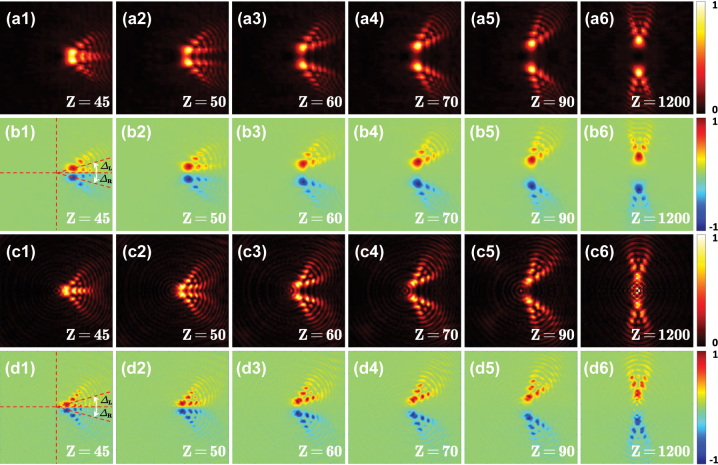
Realization of the rotational PSHE based on a single dielectric metasurface. The light intensities and the Stokes parameters *S*_3_ on the transverse plane *Z* (the metasurface at *Z* = 0) under a linearly polarized light illumination obtained using the Fresnel diffraction integral formula (the first and second rows) and the FDTD method (the third and fourth rows). The first and third rows show the distributions of the normalized light intensity. The second and fourth rows show the normalized Stokes parameters *S*_3_ corresponding to the intensities shown in the first and third rows, respectively.

Let a linearly polarized light beam normally impinges on the metasurface, and the beam intensities and the Stokes parameters *S*_3_ are calculated (see Figure 5). The obtained results come with a good agreement between Fresnel diffraction integral formula (the first and second rows in Figure 5) and the FDTD method (the third and fourth rows in Figure 5). We can observe from Figure 5 that, after passing through the metasurface, the impinging beam becomes a fan-shaped one consisting of two blades that are similar in internal structures, whose patterns look like lobes. These two lobes have the opposite spin states (blue and red denoting the RCP and LCP components in the second and fourth rows of Figure 5). As shown in Figure 5(b1) and (d1), Δ_**L**_ and Δ_**R**_ are used to characterize the rotation angle of the LCP and RCP component, respectively. Hence, the splitting angle between the two components is Δ_**L**_ − Δ_**R**_. Such spin-dependent splitting is a typical behavior of the PSHE, which results from the spin–orbit interaction. It describes the mutual influence between the spin and orbit angular momenta of light, where a spin part associated with circular polarizations and an orbital part associated with the direction of wave vector or phase front, caused by spatial polarization state manipulation [51]. PB phase is associated with local change in polarization of light, which is the quantitative representation of spin–orbit interaction. Here we realize local polarization state manipulation by constructing spatially variable nanostructures in all-dielectric metasurface. After a light beam passes through the designed metasurface, the output beam obtains an additional spin-dependent PB phase accompanied by spin–orbit interaction. Spin dependent wave-vector shifts arise due to spin-dependent PB phase gradients. The shift of wave vector in momentum space will cause the spin Hall shift in position space after propagation. As shown in Figure 5, during the post-splitting propagation, the two spin-dependent components rotate in opposite directions along the propagation path, and this behavior seems like a fan being opened with its two blades going apart from each other. The underlying physics is that the two spin components have opposite rotational phase governed by Eq. (6). The angle between the two lobes gradually increases to 180° on an observation plane locating at about *Z* = 1200 μm, and the angle keeps 180° after *Z* ≥ 1200 μm.

**Figure 6:**
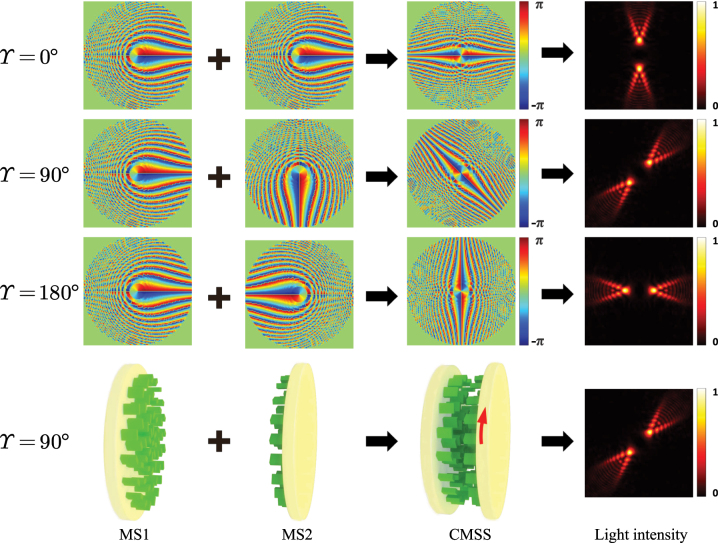
Schematic illustration of the cascaded metasurfaces with the same structure parameters to realize light beam rotation on a fixed observation plane. The first three rows show the phases introduced by the metasurface1 (MS1), metasurface2 (MS2), cascaded metasurfaces (CMSS), and light intensity distributions in the order from left to right. *Υ* is the rotation angle of metasurface2 relative to metasurface1. The last row shows the schematic of the combination of the two metasurfaces corresponding to *Υ* = 90°.

We also show that the rotation angle in the rotational PSHE are tunable using cascaded metasurfaces [52] with the same structure parameters. As in Figure 6, the adjustment can be achieved by changing the relative rotation angle between two metasurfaces. Intensity patterns of transmitted beams reaching stable states and phase distributions introduced by the metasurfaces under different rotation angles (some examples of 0°, 90°, 180°) of the second metasurface. As an example, schematic of the metasurfaces correspond to rotation angle of 90° is shown in the last row of Figure 6. Note that the two metasurfaces should be placed face to face, because the Pancharatnam–Berry phase metasurface is a spin-dependent element and inverts the handedness of the incident beam. Previous studies have reported that two cascaded diffractive optical elements or metasurfaces with similar phase profile used in this paper act as a varifocal metalens, and the focal length is tunable by a simple mutual rotation of the two elements [53–56]. The transmission functions of the two cascaded elements in Refs. [53–56] are the complex conjugate of each other, while the two cascaded metasurfaces here have the same transmission function.

**Figure 7:**
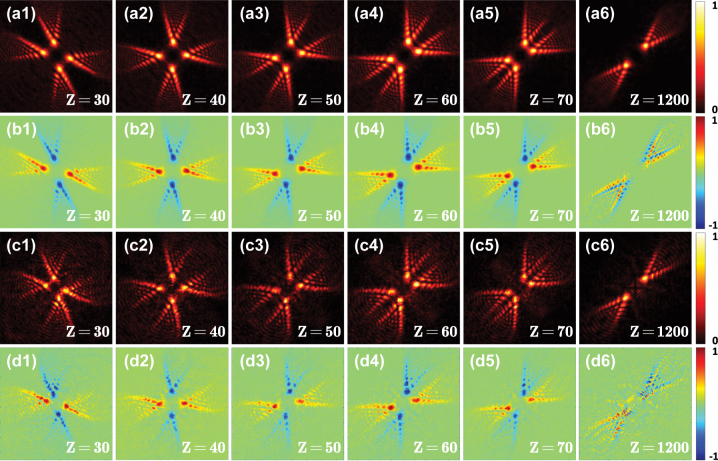
Realization of the rotational PSHE based on the cascaded metasurfaces. The light intensities and the Stokes parameters *S*_3_ of the beams generated by the cascaded metasurfaces under a linear polarization illumination obtained using the Fresnel diffraction integral formula (the first and second rows) and the FDTD method (the third and fourth rows). The first and third rows show the distributions of the normalized light intensity. The second and fourth rows show the normalized Stokes parameters *S*_3_ corresponding to the intensities shown in the first and third rows, respectively.

The simulated transverse intensities and Stokes parameter *S*_3_ of the beams generated by the cascaded metasurfaces under a linear polarization illumination are shown in Figure 7. The calculated results with a good agreement between Fresnel diffraction integral formula (see the first and second rows of Figure 7) and the FDTD method (see the third and fourth rows of Figure 7). Under a linearly polarized Gaussian beam normally impinging on two cascaded metasurfaces, the transmitted beam is also fan-shaped but consisting of four blades (see Figure 7), and these four lobe-like spin-dependent splitting patterns rotate and evolve along the propagation path. During the propagation, two of the blades actually form a subfan that looks as if it is being opened, and the other two blades form another one that behaves similarly. After propagating for about *Z* = 1200 μm, the two subfans completely overlap, which means that the pattern as a whole reaches a stable state where there appears to be only two blades. When the transmission distance is beyond *Z* = 1200 μm, the pattern no longer rotates. By comparing the results in Figures 5 and 7, we can find that the lobe number of the spin-dependent splitting generated by two cascaded metasurfaces is twice of the lobe number produced by a single metasurface before the transmitted beam reaches a stable state. However, the lobe number of the fan-shaped beam generated by either a single metasurface or two cascaded metasurfaces is finally two after the beam arrives at the stable state.

**Figure 8:**
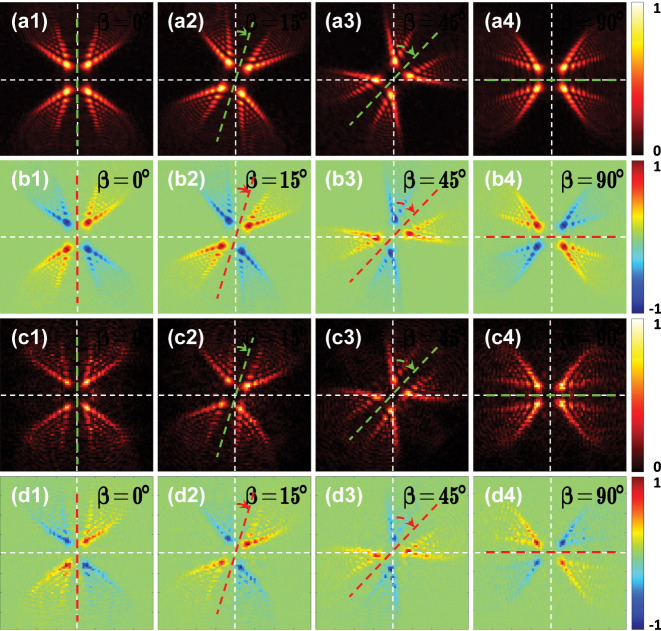
The normalized light intensities and the Stokes parameters *S*_3_ on a transverse plane *Z* = 45 μm under a linear polarization illumination for *β* = 0°, *β* = 15°, *β* = 45°, and *β* = 90° in the order from left to right, obtained using the Fresnel diffraction integral formula (the first and second rows) and the FDTD method (the third and fourth rows). The first and third rows show the distributions of the normalized light intensity. The second and fourth rows show the corresponding normalized Stokes parameters *S*_3_.

In addition, for a fixed observation plane, spin-dependent splitting pattern can be rotated by rotating the second metasurface. Four rotation angles of metasurface2 are chosen as examples, and the intensity distributions as well as the Stokes parameters *S*_3_ on the observation plane locating at *Z* = 45 μm are obtained from the Fresnel diffraction integral formula and the FDTD method. It can be seen from Figure 8 that the rotation angle *β* of the intensity pattern is half the rotation angle *Υ* of metasurface2, which implies that we can adjust the rotation angle of the splitting patterns on a fixed plane by rotating the second metasurface.

**Figure 9:**
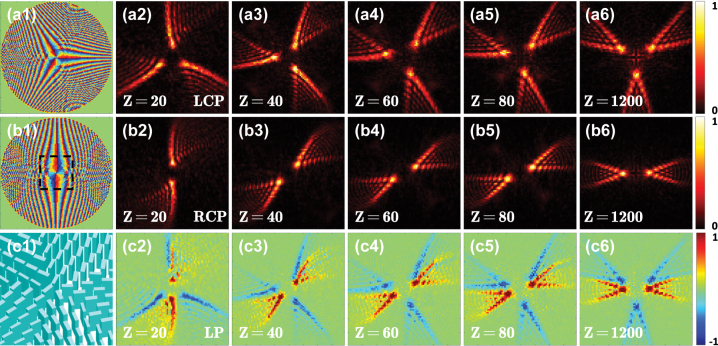
Realization of the aymmetrical rotational PSHE. (a1) The modulated phase for the LCP incidence. (a2)–(a6) The light intensities on the transverse plane at different propagation distances *Z* under a LCP light illumination. (b1) The modulated phase for the RCP incidence. (b2)–(b6) The light intensities on the transverse plane at different propagation distances *Z* under a RCP light illumination. (c1) The enlarged selected area in the black dotted square in (b1). (c2)–(c6) The Stokes parameters *S*_3_ on the transverse plane at different propagation distances *Z* under a linearly polarized light illumination.

Finally, an interesting finding is that, by introducing a dynamic phase and combining it with the original Pancharatnam–Berry phase, the lobe number of each of the splitting patterns from the metasurface can be separately controlled, which can be used to produce an asymmetrical rotational PSHE. Different than the previous discussions where we only use the fourth nanofin described in Section 2.2, the following results are obtained under the utilization of all the eight nanofins shown in Figure 2. In other words, the implementation of the (symmetrical) rotational PSHE is by changing the rotation angle of the fourth nanofin (the length and width are fixed), while the realization of the asymmetrical rotational PSHE is by changing not only the rotation angle of the nanofin but also the size of it (the eight nanofins have different sizes). Also, slightly different from Eq. (6), the modulated phases for the LCP incidence and the RCP incidence now become

(8) $$\psi_{1}^{\text{LCP}}\left(r,\theta \right)=k_{1}\cdot \text{ceil}\left(a\cdot r^{b}\right)\cdot \left(\theta +\theta_{1}\right),$$

(9) $$\psi_{2}^{\text{RCP}}\left(r,\theta \right)=k_{2}\cdot \text{ceil}\left(a\cdot r^{b}\right)\cdot \left(\theta +\theta_{2}\right),$$

where *k*_1_ and *k*_2_ control the lobe number of the splitting pattern for the LCP incidence and the RCP incidence, respectively, *θ*_1_ and *θ*_2_ represent the rotation angle at which the fan-shaped beam reaches the stable state for the LCP incidence and the RCP incidence, respectively. The values of *a* and *b* remain the same as below Eq. (6). In order to observe the asymmetry clearly, we choose the following values for the rest of the parameters: *k*_1_ = 3, *k*_2_ = 2, *θ*_1_ = 0°, and *θ*_2_ = 90°. The two modulated phases for the LCP incidence under *k*_1_ = 3 and the RCP incidence under *k*_2_ = 2 are presented in Figure 9(a1) and (b1), respectively. Furthermore, Figure 9(c1) shows the detailed arrangement of the nanofins in the black dotted square from (b1). It is clear in Figure 9(c1) that these nanostructures not only have different orientation angles but also different sizes, which is caused by us introducing the dynamic phase to the design. From the FDTD results shown in the first and the second rows of Figure 9, it can be observed that the patterns of the LCP beam and the RCP beam differ in the numbers of lobes, which are 3 and 2, respectively, corresponding to the values of *k*_1_ and *k*_2_. Similar to the regular rotational PSHE, the patterns also rotate along the propagation path, and stabilize at the distance of 1200 μm. In addition, we can see from the third row of Figure 9 that the total number of lobes for a linearly polarized beam is 5, which is the sum of the values of *k*_1_ and *k*_2_. This agrees with our intuition. By altering the values of *k*_1_ and *k*_2_, one can realize a combination of arbitrarily asymmetrical spin-dependent splitting patterns for the linearly polarized light illumination. However, if *k*_1_ and *k*_2_ are too large, the diffraction efficiency will decrease, and thus the diffracted beam intensity will be attenuated.

## 4 Conclusions

The rotational PSHE based on Pancharatnam–Berry phase metasurfaces is studied. Both the Fresnel diffraction integral formula and the FDTD method are employed, which yield consistent results. For a linearly polarized light normally illuminating a single metasurface, the beam splits into two lobes at the transmission side of the metasurface. Each of the two lobes rotates, and the angle between the two lobes changes along the propagation path. Meanwhile, the lobe number and rotation angle in rotational PSHE are both tunable by changing the polarization state of the incident beam and the mutual rotation angle between two cascaded metasurfaces, respectively. Furthermore, by introducing the dynamic phase, the lobe number for the LCP incidence and the RCP incidence can be independently controlled. The proposed scheme provides a flexible and powerful way to realize the multidimensional and active manipulation of spin photons, which may find potential applications in optical communications and optical microscopy. While the rotational PSHE is demonstrated here using metasurfaces, it can also be conveniently realized using liquid crystals, which is a future direction to achieve cost-effective multidimensional manipulation of spin photons.
